# Observing the determinants of the psychotherapeutic process in depressive disorders. A clinical case study within a psychodynamic approach

**DOI:** 10.3389/fpsyg.2015.00477

**Published:** 2015-04-21

**Authors:** Osmano Oasi

**Affiliations:** Department of Psychology, Catholic University of MilanMilan, Italy

**Keywords:** depressive disorders, personality configurations, mental functioning, psychodynamic approach, case study

## Abstract

This paper focuses on the relationship between depressive disorders, personality configurations, and mental functioning. A one-year treatment of a young man with the diagnosis of Depression is presented: the clinical and empirical points of view are described in depth through an assessment at the beginning and at one year after of an oriented psychodynamic psychotherapy. SCID I and II and HAMRS were administered to the patient in assessment phase. In the same phase he filled in BDI-II, and DEQ; the psychotherapist completed SWAP-200. These clinician instruments were used again after 1 year of the treatment. The PDM point of view is also presented. All sessions are audiotaped: 12 verbatim transcripts were coded with the Defense Mechanisms Rating Scale and CCRT. The results show a decrease in depressive symptoms, a change in some personality configurations, but a substantial invariance of the introjective profile, and a modification in mental functioning.

To S. J. Blatt and his work

## Introduction

The debate about the relation between depression and personality has been broad and has involved many questions about the mutual influence between them and their impact on outcome treatment. According to Hirschfeld et al. (1983) the connection between depression and personality has been overshadowed for a long time by three factors. First of all, by the evidence that affective disorders represent a heterogeneous diagnostic group, in which personality traits can be influenced by the type of depression and by some other variables, such as sex and age. Secondly, the lack of explicit criteria both to define affective disorders and to measure personality traits make result replicability and comparison across studies very difficult. Finally, both clinicians and researchers tend to examine their patients when they are depressed, underestimating the influence of depression on personality. Klein et al. (1993) and Widiger (1993) have described several possible relations between personality and depression: (1) personality can determine a vulnerabilty or predisposition to depression; (2) personality can be modified by depression; (3) personality and depression can reciprocally influence each other, especially in regard to their expression. Some studies (Charney et al., 1981; Koenigsberg et al., 1985; Shea et al., 1987; Black et al., 1988; Pilkonis and Frank, 1988; Pfohl et al., 1990) have highlighted that the patients diagnosed with a disease of the depressive spectrum show high levels of comorbidity with Axis II Personality Disorders (PDs) with a prevalence of borderline, schizotypal, passive-aggressive and dependent PD. Furthermore, the experience of a protracted depression can produce significant personality changes in terms of self-perception and interactive style: pessimism and dependence, for example, can become permanent personality traits (Hirschfeld, 1999). Johnson et al. (2005) found an association of specifical PDs traits (antisocial, borderline, dependent, depressive, histrionic, and schizotypal), evident by early adulthood, with a risk of the development of unipolar depressive disorders by middle adulthood. Depressed subjects with comorbid PDs are more likely to present a younger age of onset of depressive illness (Charney et al., 1981; Pfohl et al., 1990), prior hospitalizations (Black et al., 1988), less social support, and more stressful life conditions (Pfohl et al., 1984, 1990), a worse depressive symptomatology and higher percentages of suicidal thoughts and suicide attempts (Pfohl et al., 1984; Black et al., 1988; Diguer et al., 1993).

A first position about the possible correlation between PDs and depression is presented in different research studies (Patience et al., 1995; Ramana et al., 1995; Casey et al., 1996); in particular some suggest that the PDs adverse effects can be observed especially in depressed patients characterized by comorbidity with more than one PD (O’Leary and Costello, 2001) or with particular types of PDs, such as the Cluster A ones (Sato et al., 1994). Hirschfeld (1999) maintains that depression can be secondary to the presence of PDs and that patients diagnosed with avoidant, borderline or histrionic PD, particularly sensitive to frustrations, are more likely to become disforic or depressed. Chronic depression can lead to relational deficits and working problems; in these situations the criteria for PDs can be satisfied and an effective treatment for depression can also alleviate personological symptomatology. A meta-analysis of Newton-Howes et al. (2006) reported a doubling of the risk of a poor outcome for patients suffering from comorbid PD with depression compared with no PDs ones. The same authors with other colleagues confirm this position in their updating systematic review and meta-analysis (Newton-Howes et al., 2014).

Nevertheless, a complete overview of literature shows that different studies do not support this point of view about correlation between affective disorders and PDs. For example, although PDs are generally supposed to produce negative effects on depression outcome, Mulder (2002) asserts that the best-designed studies reported the least effect of personality pathology on depression treatment outcome, suggesting that comorbid personality pathology should not be seen as an impediment to good treatment response. In fact, the influence of personality on depression outcome depends on different factors: (1) the rate of personality pathology varies markedly depending on how it is measured; (2) depressed patients with personality pathology appear less likely to receive adequate treatment in uncontrolled studies; (3) finally, studies rarely monitor for depression characteristics (e.g., chronicity, severity) that may influence outcome and be related to personality pathology. Pointing in the same direction, Blom et al. (2007) showed that depression treatment outcome was associated with symptom severity and duration rather than personality variables.

As has previously been noted, this debate about the relations between depression and personality has had an important effect in the evaluation of the treatment of this syndrome. The first important research program about the effectiveness of the psychotherapy of depressive disorders is the Treatment of Depression Collaborative Research Program (TDCRP), sponsored by the National Institute of Mental Health (NIMH). Four treatment conditions at three different research sites were designed: Cognitive Behavior Therapy (CBT), Interpersonal Psychotherapy (IPT), pharmacological condition (Imipramine) plus clinical management (IMI-CM), and pill placebo plus clinical management (PLA-CM). Two hundred and forty depressed outpatients were randomly assigned to the four treatment conditions (Elkin et al., 1985). Initial results suggested the IPT and antidepressant drugs might superior to CBT with more severely depressed patients groups (Elkin et al., 1989). Some years after, some authors (Elkin et al., 1996; Jacobson and Hollon, 1996; Hollon et al., 2002) hypothesized that in across sites the psychotherapist’s expertise made a greater difference determining the gaps with other kind of treatments (in particular, in favor of IPT) as well as medications in relation with severity of depression. The Ablon and Jones’s (1999) study, working on the transcripts of treatment sessions of TDCRP, found that both CBT and IPT had closer adherence to the cognitive-behavioral prototype that produced more positive correlations with outcome measures, than to the reference model. These overlaps were confirmed by other studies: CBT, IPT, combined or not with medication, should have similar outcomes (Quilty et al., 2008; Peeters et al., 2013). Other interesting studies based on TDCRP have pointed out the attention about the contribution of the therapists in the psychotherapeutic process, discovering their influence in the outcome results (Blatt et al., 1996). Further analyses of data have highlighted significant treatments differences at the 18-month follow-up in the life adjustment of the patients: in particular, the patients after both IPT and CBT treatment reported greater effects on their ability to establish and maintain interpersonal relationships and to recognize the sources of their depression and to prevent it than control groups (Blatt et al., 2000). Finally, other researches (Blatt and Zuroff, 2005; Carter et al., 2011) have underlined the need to take in consideration not only the quality of the relationship, but also the patient’s pretreatment personality configurations. These considerations are very important for this study.

Although there are many successful treatments of depression employing psychodynamic approach (Abraham, 1911; Asch, 1966; Jacobson, 1971; Arieti and Bemporad, 1978; Stone, 1986), in this kind of treatment the diagnostic criteria and the symptomatic improvement are not well documented, neither it has been demonstrated in placebo-controlled trials to be effective. Generally speaking, the psychodynamic approach is useful both in severe and mild depressive conditions, combined, or not with specific pharmacotherapy (Gabbard, 2000). In this direction, some recent studies have attempted to assess the impact of psychodynamic treatments for different depressive disorders systematically. Gallagher-Thompson and Steffen (1994), for instance, found that psychodynamic psychotherapy was comparable to CBT in reducing depressive symptoms in the caregivers of elderly family members. Shapiro and colleagues (Shapiro et al., 1994, 1995) compared CBT with IPT in a randomized, controlled trial for depression and found them to be equivalent in efficacy. Hilsenroth et al. (2003) found a significant reduction in depressive symptoms with psychodynamic treatment and a decrease in symptoms correlated with the use of psychodynamic treatment techniques. Busch et al. (2004) suggested that focused psychodynamic psychotherapy could be a valuable adjunct for the treatment of depression, including the vulnerability to recurrence of depression, and in some cases it may be effective alone. On the basis of their clinical experience, the authors recommend this approach primarily in patients with mild or moderate major depression and dysthymic disorder (DD). Recent publications (Luyten and Blatt, 2012; Luyten, 2014) have highlighted the effectiveness of psychodynamic treatment of depression: in particular, as compared to long term outcomes, no great difference has been found between CBT and psychodynamic therapy. On the other hand, the results reported by Jakobsen et al. (2011a,b) are not very encouraging, as there are no findings supporting or refuting the effect of Interpersonal or Psychodynamic psychotherapy, neither Cognitive therapy, compared with “treatment as usual” for patients with major depressive disorder (MDD).

Probably, as Rush and Thase’s (1999) review suggested some years ago, to think that each psychotherapy approach supplies worse or better results in relation to different kinds of depressive disorders [MDD, DD, D-NOS (Depression not otherwise specified), or D with GMCs (Depression associated with (not physiologically caused by) general medical conditions)] could be the best position. Indeed, the contribution of medications needs considering, above all in depressive disorders because the biological and genetic components are not irrelevant part of the psychopathological development both in Unipolar Depression and Bipolar Depression (Power, 2013). In this perspective, it could be very useful integrate psychotherapy and pharmacotherapy.

An important point of view for evaluating the effectiveness of the treatment on empirical point of view within the psychodynamic approach is represented by the study of defense mechanisms (Perry et al., 1993, 1998; Perry and Kardos, 1995). Bond and Vaillant (1986) found that patients diagnosed with major affective disorder have significant relationships with specific defense style. Mullen et al. (1999) evaluated the issue of stability of defensive functioning and personality organization over the course of psychiatric illness by examining a large sample of well-characterized outpatients with a DSM-IV determined diagnosis of MDD, over a defined course and period of treatment. They observed a significant decrease in “maladaptive” defenses in the entire sample, while “image-distorting” and “self-sacrificing” defenses did not change significantly. Interestingly, “adaptive” defenses remained unchanged from baseline and a progressive increase in the usage of mature defenses in a group of patients diagnosed with major depression is observed, while no changes were observed in the neurotic ones levels. In a literature review on this issue Bond (2004) reported that adaptiveness of defense style was associated with mental health and that some diagnoses were characterized by specific defense patterns: in particular, depressive symptomatology was found to be positively correlated with the use of immature defense styles and negatively correlated with the use of mature ones in comparison with controls, while anxiety disorder patients tended to use more neurotic and immature defenses than non-patients. Bond’s (2004) conclusions were corroborated through a meta-analysis study conducted in which Calati et al. (2010) assessed through the three-factor DSQ versions two different psychiatric diagnoses, MDD and Panic Disorder (PD), to evaluate their potential specificity of defense styles. Perry and Bond (2012) observed the change in defensive functioning of a group of patients with depressive, anxiety, and/or PDs in long-term dynamic psychotherapy largely followed the hierarchy of defense adaptation. In particular, the lowest (action) and the highest (high adaptive) defense levels improved significantly, as did overall defensive functioning (ODF), that still remained below the healthy-neurotic range. Generally speaking, the use of defense mechanisms and their relationship with psychopathology and change (Bond, 2004) is an important point of view for the assessment and the evaluation of the psychotherapeutic process. In particular, as the Psychodynamic Diagnostic Manual (PDM Task Force, 2006) reminds us, they represent a way of evaluating the mental functioning (M axis) of the patient.

## Aims and Hypotheses

Aim of this work is to identify a possible interaction among personality, mental functioning, and clinical syndrome by a comprehensive assessment (Serretti et al., 2007) of one depressed patient. The case of Mr. F is presented, assessing the subject’s psychotherapeutic psychodynamic process in the context of his personality structure, to evaluate the possible correlation between a defensive functioning evolution and a significant symptom change during the course of the psychotherapy. The hypothesis is to observe a changement in Mr. F’s depressive symptomatology and mental functioning, through a different configuration of personality features and an evolution from primitive to mature defensive mechanisms (Akkerman et al., 1999; Bond and Perry, 2004).

An additional aim is to illustrate the integration of a qualitative approach based on the therapist’s perspective, and a quantitative approach based on data gathered using empirical instruments and statistical methods. To bridge the gap between clinicians and researchers and to show a clinically sophisticated and empirically grounded practice in psychodynamic framework inspired this paper (Kazdin, 1982; Moran and Fonagy, 1987; Fonagy, 1993; Wallerstein, 1995; Roth and Fonagy, 1996; Shedler, 2002; Porcerelli et al., 2007; Kächele et al., 2009; Levy et al., 2012). In this perspective it is important to choose well-validated empirical instruments: a description of these is proposed below.

## The Case of Mr. F

Mr. F is a 26-year-old man self referred to the Community Mental Health Center near Milan in November 2012. He had already been followed by the other Psychiatric Service in 2006 when he was working in a touristic mountain location, where he started to perceive fatigue and sadness. The Service there put diagnosis of Social Phobia and Obsessive–Compulsive PD.

Mr. F had already mentioned family problems, concerning in particular his mother’s family of origin. Following a car accident Mr. F had to return to his family in 2007. Since then he had been followed with antidepressant until May 2010. In this period he enrolled at the evening technical school, did some work as metalworker, took the antidepressants regularly and did some psychological sessions. Then he returned himself again in the same Community Mental Health Center in November 2012: he is not working – he had resigned –, he had spent a few weeks in a Religious Community and is now living at home “vegetating.” In January 2013 he started a psychodynamic psychotherapy once a week. A pharmacological therapy arranged with antidepressant drug (duloxetina, 60 mg die) by psychiatrist. It is very important to note that the pharmacological therapy was preserved without any modification during the year of the psychotherapy. The pharmacotherapy compliance has been good.

## Methods and Measures

### Method

A “practice base” approach has been used (Fonagy and Moran, 1993; Margison et al., 2000; Castelnuovo, 2010): the data of a psychotherapy carried out in a Psychiatric Service was collected at different times during 1 year of a psychodynamic treatment (see table of research plan in **Table 1**). A clinical case study was formulated.

**Table 1:** Research plan.

		Month of treatment											
	Assessment phase	1st	2nd	3th	4th	5th	6th	7th	8th	9th	10th	11th	12th
SCID I and II	X												
BDI II	X												X
HDRS	X												X
DEQ	X												X
SWAP-200			X										X
DMRS		X	X	X	X	X	X	X	X	X	X	X	X
CCRT			X					X					X

### Assessment Measures

#### The Structured Clinical Interview for DSM-IV, Axis I and II – SCID-I and SCID-II

The Structured Clinical Interview for DSM-IV Axis I Disorders (First et al., 1997a) is a semi-structured interview used for evaluating some of the clinical symptoms described in DSM-IV in Axis I. It correctly evaluates affective disorders, schizophrenia, and other psychotic disorders, such as substance-related disorders, anxiety disorders, somatomorphic disorders, eating disorders, and adaptive disorders.

The Structured Clinical Interview for DSM-IV Axis II PDs (First et al., 1997b) is a semi-structured interview used for evaluating different PDs described in the DSM-IV from the categorical approach to determine the actual diagnosis. Moreover, each question has four possible answers to choose from, which also allows a dimensional approach.

#### The Hamilton Depression Rating Scale – HDRS

The Hamilton Depression Rating Scale (HDRS) o HAM-D (Hamilton, 1960) is a 21-item screening instrument designed to measure the severity of illness in adults already diagnosed as having depression. The first 17 items are the nuclear items for depression and on these is based the severity cut off: >25 severe depression; 8–24 mild depression; 8–17 light depression; <7 no depression.

The HAM-D is one of the most widely used instruments for measuring outcome in mood disorders, and it offers high validity and reliability in measuring response to treatment. The HAM-D is administered by a clinician or health care professional during or immediately following a client interview and takes approximately 15–20 min to complete, depending upon the interview structure.

#### The Beck Depression Inventory – BDI

The Beck Depression Inventory (BDI) is a self – report questionnaire to measure depression symptoms and severity. The BDI II (Beck et al., 1996), published in 1996, is a substantial revision of the original version of 1961. It was developed to match to DSM-IV criteria for diagnosing depressive disorders and included items measuring cognitive, affective, somatic, and vegetative symptoms of depression. Twenty-one items are evaluated on a 4-point scale that indicates degree of severity. The recall period for items is the last 2 weeks and the total score can go from 0 to 63 points. The BDI II suggests four different cutoff for different degree of depression: 0–13 minimal range; 14–19 mild depression; 20–28 moderate depression: 29–63 severe depression.

Also BDI II is one of the most widely used instruments for measuring outcome in mood disorders, and it offers high validity and reliability in measuring response to treatment as HDRS. Self-administration is 5–10 min; special attention is requested by item 9 (suicide ideation) and item 2 (hopelessness) in the scoring process.

#### Shedler–Westen Assessment Procedure – SWAP-200

The Shedler–Westen Assessment Procedure (SWAP-200; Westen and Shedler, 1999a,b) is a set of 200 personality-descriptive statements. The clinician describes the patient by arranging the statements into eight categories, from those that are not descriptive (pile 0) to those that are highly descriptive of the patient (pile 7), giving each item a score from 0 to 7. Items are written in straightforward language without recourse to jargon. The tool is based on the Q-sort method that requires clinicians to arrange items into a fixed distribution. The statements have been developed from the theoretical and empirical literature on personality and PDs, defense mechanisms, DSM-III and IV. From the 30 statements that are scored higher the case formulation is obtained and takes into account the three main domains described by the SWAP-200: (1) motivations, ideals, anxieties and conflicts; (2) psychological resources; (3) experience of the self, of the others, and relationship between the patient and the others.

The SWAP-200 assessment leads to two kinds of diagnosis: the first expressed in PD factors, that are descriptions of the patient’s personality with the kind of Axis II – DSM-IV disorder; the second expressed in Q factors that are descriptions of the patient’s personality through its degree of similarity (proximity) to 11 prototype personality empirically derived styles. PD and Q factor scores are expressed in *T* scores: the cutoff to determine the presence of a PD is 60, while a score to ≥55 indicates the presence of personality traits of a specific PD, but under clinic threshold. No diagnosis of PDs is done, if the high functioning factor is ≥60.

#### The Depressive Experience Questionnaire – DEQ

The Depressive Experience Questionnaire (DEQ; Blatt et al., 1976) is a self – report questionnaire to differentiate between dependence and self-criticism, related to greater risk of psychopathology in general and of depression in particular. It is a 66 item questionnaire evaluated on a 7-point Likert scale, from 1 (strongly disagree) to 7 (strongly agree). The scoring leads to three scales: dependence, self – criticism and efficacy. Since the Efficacy scale does not measure a Blatt’s theoretical concept, is less important than the other two scales that measure, respectively, the anaclitic and introjective depression.

### Process Measures

#### The Defense Mechanisms Rating Scale – DMRS

The Defense Mechanisms Rating Scale (DMRS; Perry, 1991) manual describes how to identify 27 defense mechanisms in video or audiotaped sessions or transcripts. The manual includes a definition of each defense, a description of how the defense functions, a section on how to discriminate each defense from similar ones and a three point scale to identify the absence of the defense (0), the probable use (1) and the definite use (2). The scale allows three different ways to assess defenses: Individual Defense Score, Defense Level Score, and ODF score. In clinical samples scores usually range between 2.5 and 6.5.

The defense mechanisms are hierarchically arranged in seven clusters, from the more primitive to the more mature: (1) Acting: acting out, passive aggression, help rejecting complaining. (2) Borderline: splitting of others’/self image, projective identification. (3) Disavowal: negation, projection, rationalization. (4) Narcissistic: omnipotence, idealization, devaluation. (5) Neurotic: repression, dissociation, reaction formation, displacement. (6) Obsessive: isolation, intellectualization, undoing. (7) Mature: affiliation, anticipation, humor, self assertion, self observation, sublimation, suppression.

#### The Core Conflictual Relationship Theme Method – CCRT

The Core Conflictual Relationship Theme Method (CCRT; Luborsky, 1984) for research purposes is applied to text parts, obtained from the transcription from audiotaped sessions, in which the patient recounts about his/her interactions with significant others. These “Relational Episodes” (RE) are characterized by a specific narrative structure and can be referred to a current or past episode, really happened or just dreaded or expected. They can concern the patient’s relationship with himself or with others, including the therapist. The core conflictual relationship theme is formulated through different steps: (1) The RE are identified in the session transcripts. (2) The patient’s desires, needs, and intentions (W), the relational partner’s responses (RO), and the patient’s reactions to these responses (RS) are identified in each RE. (3) Each RE is scored using “standard categories” or “tailored categories.” (4) CCRT formulation. From at least 10 RE the most representative categories of W, RO, and RS are chosen.

## Procedures

In November 2012 Mr. F was assessed by the psychiatrist and the psychologist of the Community Mental Health Center. They confirm the previous diagnosis of Depression, made in the first contact of the patient to the same psychiatric service (from 2007 until 2010 he was followed only by a psychiatrist). After this new contact, a once a week psychodynamic psychotherapy was proposed to the patient and he accepted it. The psychotherapeutic treatment duration expected in this Department of Mental Health is generally 1 year. The psychotherapist is a middle age man, who has collaborated with this Department for more 10 years. He is member of the Italian Psychoanalytical Society. The consent for the treatment and for the data collection was obtained in advance.

Mr. F was assessed by the psychologist of the Community Mental Health Center with the SCID I and II, the HAM-D, and the DEQ. After some sessions (see **Table 1**) the psychotherapist completed the SWAP-200. The sessions were audiotaped and transcribed. On 12 monthly sessions the DMRS and the CCRT was evaluated. Both these instruments required the evaluation by two different couple of raters to obtain the inter-rater reliability. Each rater was blind to the results of the other rater. For DMRS two different clinical psychologists who received specific training about this instruments obtained a mean of Cohen coefficient kappa very good (*K* = 0.81). For CCRT two members of the Italian Psychoanalytical Society who were very expert in the Luborsky’s signature obtained a value very similar (*K* = 0.76). After one year of treatment Mr. F was assessed again with HAM-D, DEQ by the psychologist of the Community Mental Health Center and the SWAP-200 completed another time by the psychotherapist.

To improve the comprehension of the development of the treatment the therapy was divided into three phases. Phase 1: from January 2013 to April 2013; phase 2: from May 2013 to August 2013; phase 3: from September 2013 to December 2013.

## Results

### Mr. F at the Beginning of the Psychotherapy – Clinical Perspective

Mr. F appears quite dull, looking more his actual age. He is not integrated into family life at all nor in the social one. He seems introvert, not willing to expose himself, especially in the relationships, and is very isolated. His only passion is mountain biking. Soon a very marked sensitivity to criticism emerges: in everything he does there is always something wrong. For this reason he decided to withdraw from the world. In general he seems stuck, not able to move. The person Mr. F feels most hostile to and critical of is his mother. He speaks a better relationship with the father who, according to him, made the biggest mistake of his life marrying the mother; but he survives thanks to his job that keeps him away from home. Another victim of the mother seems to be his sister who, like Mr. F, had already needed psychological support. Sometimes he focuses on the theme of euthanasia, legal in other countries than Italy, like Swiss and Norway where he threaten to go, causing great worry among the family. It seems there is a strong aggressiveness towards the self in a family environment perceived as little welcoming and containing.

In the first phase of treatment Mr. F appeared closed, not very available in calling his assumptions about the world and his family into question. His functioning seems pretty much projective: others have never understood him and have expectations he knows he cannot fulfil. Still Mr. F is sure he has qualities, especially related to sports, but no one ever trusted him. This leads the therapist to wonder about whether the patient could have trust in him and in the psychotherapeutic treatment and the fear of Mr. F of been considered inadequate by the therapist also.

Under a depressive surface some personality traits did emerge in the therapy. Mr. F seems sheltered in a narcissistic position, untouchable because keeps out the confrontation with other. Emotional control is very strong and leads to a kind of “flattening” that makes Mr. F abulic, but under which there is an underneath aggressiveness represented in an hostile passivity.

### Mr. F at the Beginning of the Psychotherapy – Empirical Perspective

Within a descriptive diagnosis (American Psychiatric Association, 2000) Mr. F presents in Axis I a MDD (F32.1) – primary diagnosis – with attenuated onset probably in 2005, and Social Phobia (F40.1) – secondary diagnosis. In Axis II Mr. F presents criteria for the Avoidant PD (F60.6) and the Passive–Aggressive PD (In Appendix B of DSM-IV TR). Into a psychodynamic diagnosis (PDM Task Force, 2006) Mr. F is well described by the Personality Depressive Disorders, introjective type (P107.1), with moderate limitations and alterations of mental functioning (M205) and symptomatic pattern belonging both to Depressive Disorders (S304.1) and Adaptation Disorders (S301).

The HAM-D shows a score of 28, which indicates a severe depression; the BDI II shows a score of 25, which indicates a moderate depression. The PD scores of the SWAP-200 show at the beginning (T1) of psychotherapy a significant elevation of the Schizoid PD (*T* = 62,03) and the Avoidant PD (*T* = 60,81). Moreover, significant traits of the Schizotypal (*T* = 59,40), Borderline (*T* = 54,28) and Dependent (*T* = 53,08) disorder are present. The High Functioning score is low, under 50. The *Q* scores profiles confirm a significant elevation in the Schizoid PD (*T* = 60,44) and in the Avoidant PD (*T* = 62,46); also the Emotional Dysregulation Disorder (*T* = 64,55) is above threshold.

In respect of dimension scores of the DEQ at the beginning of the therapy of Mister F, the results show a prevalence of Self Criticism (0,66) compared with dependency (–0,59) and Self Efficacy (–1,13). An introjective depression emerges, with the prevalence of self-criticism compared with dependence. This negative value in Self Efficacy dimension is particularly remarkable and going along with the general low functioning of the patient.

### Following Treatment: DMRS, CCRT, and Comparison SWAP/DEQ

In this section the results of the trend of the psychotherapy process in a year is illustrated through three different point of view: defensive functioning, typical interactions with significant others, and features of personality in connection with some possible modifications of the depressive experience.

**FIGURE 1:**
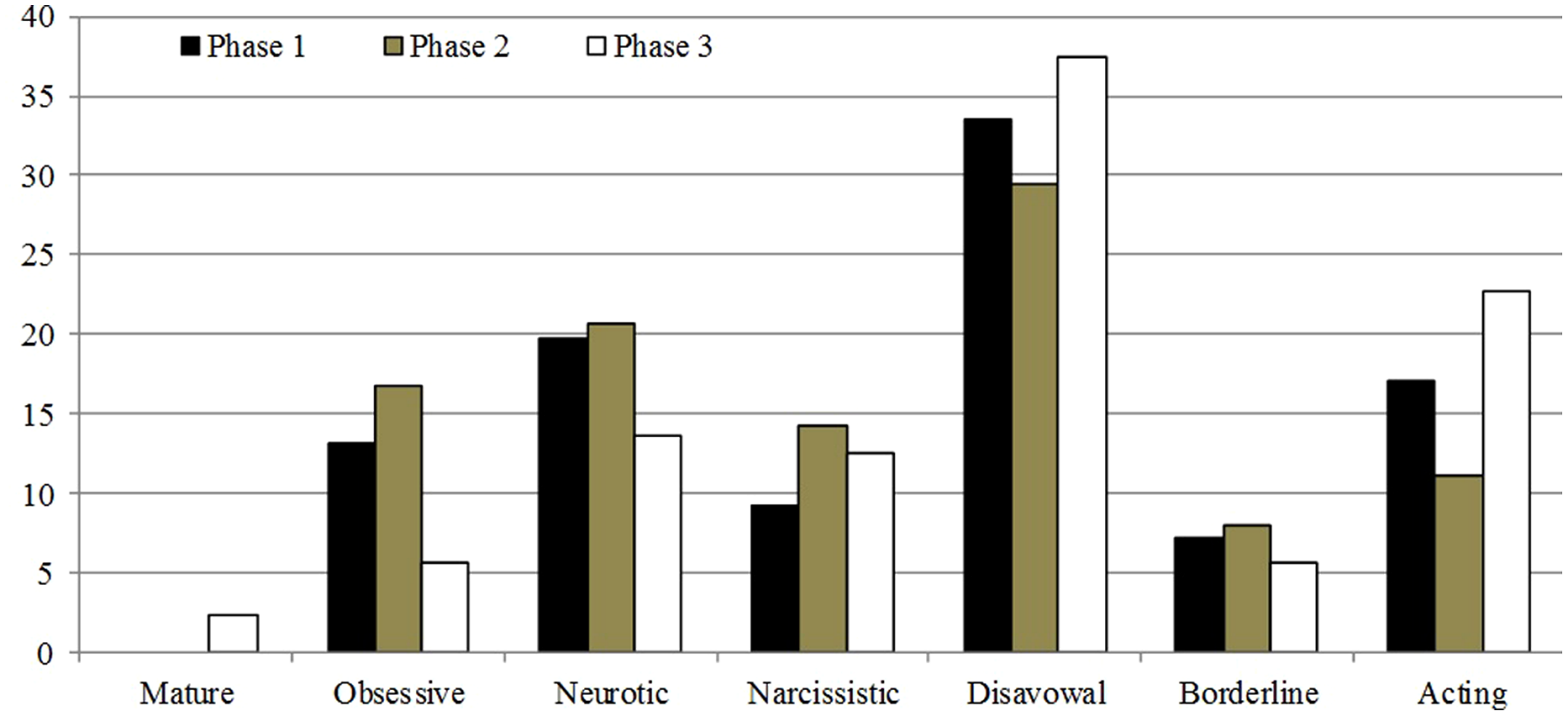
**Mr. F’s the Defense Mechanisms Rating Scale (DMRS): percentages of defensive trend in the three phases of the treatment**.

The partitioning in to three different phases of psychotherapy (see before) allows a clear illustration of the changes in the defensive functioning following DMRS scores. In general, all clusters of defense decrease during the treatment (from first to third part of it), with the exception of Mature and Acting clusters (in this specific case the level is lower compared with the first phase of psychotherapy, but higher compared with the second phase). Special attention was given to the prevalence of Disavowal defenses as main manner of the patient to take on the inner and external conflicts, and correlated emotions. In the same way, it seems important to note the appearance of the Mature defenses in the third part of the treatment (**Figure 1**).

**FIGURE 2:**
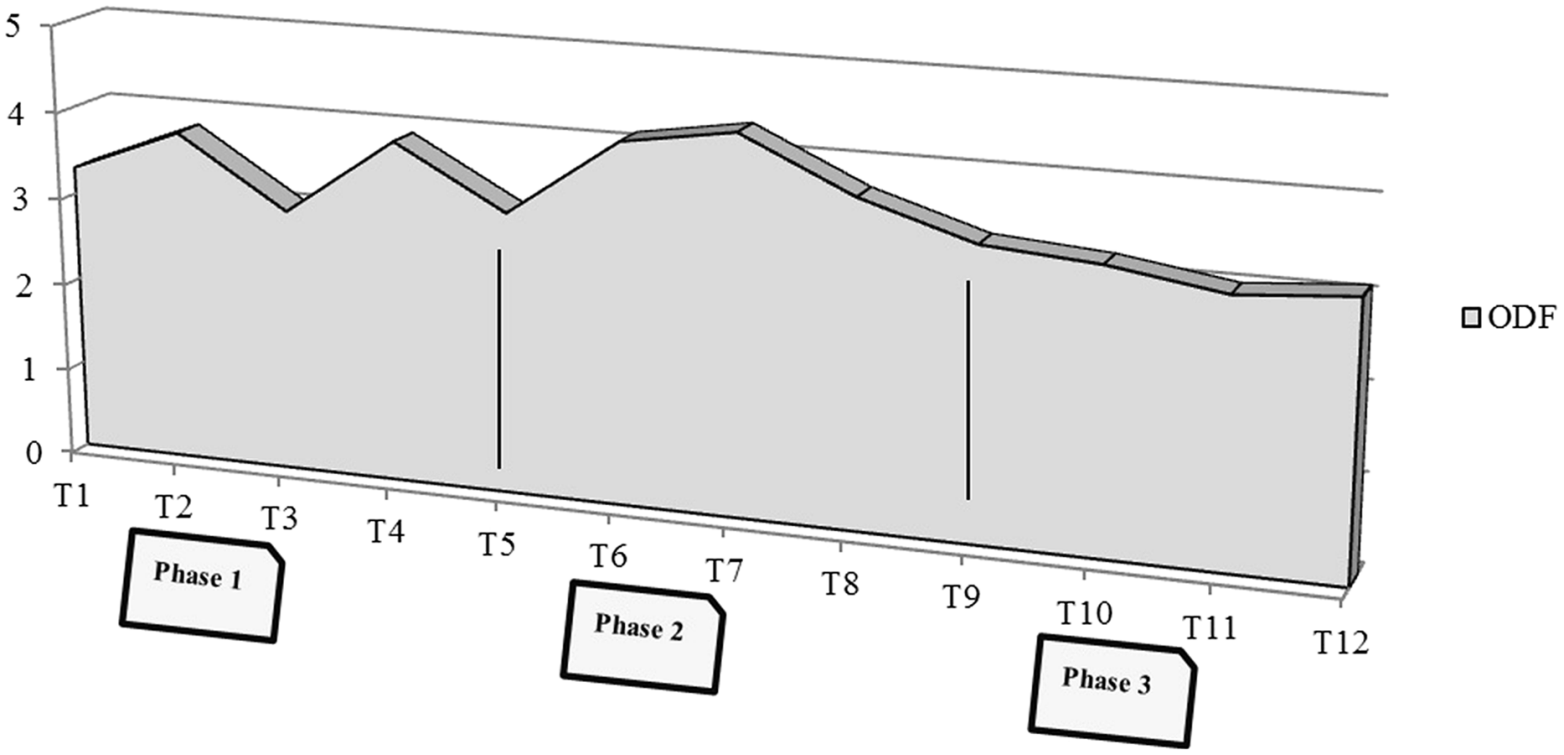
**F’s DMRS: course of the global scores of the defenses during the therapy (monthly acquisition)**.

A last remarkable result concerns the general trend of defense mechanisms (ODF index) in the one year of treatment. In the first phase of the psychotherapy an important fluctuation in the use of defense mechanisms is present (mean value = 3,5). The patient shows the highest level of the use of defense mechanisms in the middle phase of the psychotherapy (mean value = 3,8), probably at the time of the transformative psychological work. In the last phase of the year, there was a stabilization in the defensive functioning, at still clinical and rather low level (mean value = 3,2; **Figure 2**).

The same partitioning in to three phases of one year of psychotherapy is useful for showing the typical interactions with significant others through CCRT. In particular two RE for each phase are extracted: the first in relation with the other significant; the other in relation with the psychotherapist. The usual sequence in three different steps is reported: wish expression from the patient (W), responses from the other (RO), and subsequent response on the self from the patient (RS). After each example a short comment in psychodynamic perspective is reported (**Tables 2A–C**).

**Table 2A:** Mr. F’s CCRT – phase 1.

CCRT – Phase 1	
Example	Comments
P: //^1^ W (6, 7) I always wondered what I’m doing here, obviously I go on, staying here does not make me either hot nor cold.// RO (5, 4) Let’s say that with my aunt and my mom are like that, they have no confidence in me.// RO (5, 4) And then they tell me that they do not know, tell me that I do not listen. They told me, especially my mom told me that it makes no sense. When you do something nothing has ever sense then.// RS (7, 6) I do not know tomorrow, tomorrow morning I wake up, if I wake up I’ll see what there is to do! Tomorrow we’ll do: better to leave now that tomorrow, maybe by mistake you will make a family, you will make a son or maybe you will have to look after a child, “Dad is not there, why? For what?” Phase 1. Example 2. P: // W (7, 8) I’m trying to make sense why I am here, on this earth.// T: // RO (6, 8)…That perhaps you can’t find it in your parents at this time, is not it?// P: // RS (6, 7) Probably this is an unanswerable question, let’s go forward while we’re here.// T: // RO (6, 8) Why? Do you feel useless?// P: // RS (7, 6) If I have to stay here to ruin the lives of people. I did not ask to be born, I was born and then they told me it was my fault!//	Generally speaking, the desires, needs, and intentions of the patient are frustrated. Nobody, not even the psychotherapist, can be helpful for him. Since maternal figure was probability missing, any function of empathic mirroring of the other was absent; any mechanism of identification toward to the paternal figure was lacking. The patient’s reactions include anger and hate, mainly focused on own self.

^1^The // define the sentence unit codificated by CCRT.

**Table 2B:** Mr. F’s CCRT – phase 2.

CCRT – Phase 2	
Examples	Comments
1. P: // RO (4, 5) According to my mum, I ran away from problems.// W (2) You have to see who is right between you and me, if I’m right or wrong!// RS (7) If she’s right I will leave, but I won’t leave home, I will go into the other world!//	Also in this second phase of the treatment the relationship with the significant other (mother in particular) is characterized by heavy conflicts, with a regression in the mental functioning as the acting out tendence shows. On the contrary, in the clinical setting the patient proceeds to show a more willingness toward emotional validation and meaningful openness concerning the future implied by the psychotherapist interventions.
2^2^ P: // W (1, 3) When I’m wrong it is always my fault, when you do the right thing it is also about them and now we have to make choices, I put them in the field of choices: either they give me a hand or assume their responsibilities.// T: // RO (8) No, I’m saying that you are still in a phase where your parents have got it all wrong: they did not think, they have not loved, they have not invested in you. [...] If you will stay still in this position, the effort is to create continuity in the sense that you have never been used to invest in yourself, and you are affected by this, right? You don’t seem very pleased.// P: // RS (1) Yes, I am.// T: // RO (8) I wonder if it is worth pondering whether there are alternative ways of thinking. Many decide to have a different life, and you still seem a bit oriented on several fronts, right?// P: // RS (1) Yes, I should always have alternative choices.//

^2^In the following interaction the RE is playing not directly with the psychotherapist, but with his holding and mentalizing role.

**Table 2C:** Mr. F’s CCRT – phase 3.

CCRT – Phase 3	
Examples	Comments
1. P: // W (1) Some time ago I made plans, now I should think th I am here, I do not know tomorrow. Rather than begin to do something that tomorrow we will leave there, it is better to do nothing.// RO (2) What is hard is the fact that others tell you what you must do.// […] RS (6, 7) I am forced, if you don’t do what they tell you, they will leave you in the middle of a road./ RS (4, 7) I will see how far they resist: I’m going to do something, but they also will have to put something on the plate.//	In the third phase of the treatment a different reaction of the patient in relation to the psychotherapist’s interventions is more evident: the psychotherapeutic relationship built up in the clinical setting gives the patients an opportunity to experiment very different feelings coming from the other and focused on the self. The patient’s “new deal” could start from a “blank space” mentioned by Mr. F On the contrary, his inner perceptions about the situation outside of the clinical setting are unchanged after one year of psychotherapy.
2. P: // W (7) Doing the same thing for several days is boring, apart from staying outdoors. I could only give you a hand with regard to practical work, nothing more. // T: // RO (6) How do you evalute yourself, if you were to do a profile as actually used in social network? P: // W (7) I leave blank space.// T:// RO (8) This blank space has always been blank or have you decided to make it blank?// P:// RS (5) Probably I have made it blank lately T:// RO (8) We could say that by leaving it blank no one could say “why have you left that thing so!”// P:// RS (5) Bah, to the utmost they could ask you why it is blank, they always find something to say.//

In order to evaluate the features of personality in connection with some possible modifications of the depressive experience a detailed comparative analysis between the most significant DEQ and SWAP-200 items was carried out for Mr. F’s in T1 and T2 assessment profiles. A selection is made by choosing exclusively the DEQ items loading for one of the three factors (Efficacy, Self-Criticism, Dependency; Blatt et al., 1995); regarding to the SWAP-200, only the most descriptive items of the Q-factors/personality styles (Shedler et al., 2014) characterizing Mr. F’s assessment (Schizoid, Avoidant, Emotional Dysregulated, and Hostile-Externalizing) are taken into consideration. In particular, the items of the DEQ and the SWAP-200 having obtained the highest scores (from 5 to 7) or having shown a significant change (increase of more than 2 points) from T1 to T2 are focused. In this way the features of personality in connection with some possible modifications of the depressive experience are highlighted.

**Table 3** reports possible “matching” of some couples of significant items belonging to the two instruments, observing a semantic overlap between the respective statements. In addition, in some cases the scoring variations of the matched items are very similar along the therapeutic course. It is remarkable that the self-criticism is the depressive dimension that is most present in connection with the two different Q Factors/Styles of Personality (Avoidant and the Emotional Dysregulated).

**Table 3:** Comparison of some more descriptive items DEQ and SWAP-200 during the first year of the therapy.

DEQ						SWAP-200						
Item	Factor			Score		Item	Q factors				Score	
	Efficacy	Self-Criticism	Dependency	T1	T2		Avoidant	Emotional Dysregulated	Hostile-Externalizing	Schizoid	T1	T2
14. I enjoy sharp competition with others	X			5	4	8. Tends to get into power struggles			X		0	5
64. I tend to be very critical of myself		X		5	5	91. Tends to be self-critical; sets unrealistically high standards for self and is intolerant of own human defects	X				5	3
13. There is a considerable difference between how I am now and how I would like to be		X		3	5	54. Tends to feel s/he is inadequate, inferior, or a failure	X	X			7	6
16. There are times when I feel “empty” inside.		X		5	5	90. Tends to feel empty or bored		X			7	5
17. I tend not to be satisfied with what I have		X		5	4	56. Appears to find little or no pleasure, satisfaction, or enjoyment in life’s activities	X	X			7	5
34. I find it very hard to say “no” to the requests of friends.			X	5	4	199. Tends to be passive and unassertive	X			X	5	5
65. Being alone doesn’t bother me at all			X	3	5	104. Appears to have little need for human company or contact; is genuinely indifferent to the presence of others				X	4	7
11. Many times I feel helpless			X	5	5	127. Tends to feel misunderstood, mistreated, orvictimized.		X	X		6	7

### Mr. F at the End of the Psychotherapy – Clinical Perspective

Half-way through the first year of his therapy Mr. F began to show some first variations in his mental functioning that could be considered as possible changing signs.

In particular, his aggressiveness is sometimes canalized in different ways, taking more “mobile” forms: for example, the patient’s political involvement, arising from a generalized accusation against politicians, considered unable to understand the citizens’ real needs, evolves into his activism within the Five Star Movement. Mr. F identifies himself with different Beppe Grillo’s Movement program points, first of all with the “citizenship income” principle, because he would take great advantage of it. However, it’s vain to highlight this one and other secondary “advantages”: he asserts living “from day to day,” helping both his father and his uncle (his father’s brother) to be accepted by his family. The subject’s sport engagement increases (races of running and swimming). He is an active member of the Youth Council in his town, where he puts effort into the organization of social aggregation events (in particular the Beer Party) with little or no returns. These seem to be different ways in which Mr. F tries to sublimate or to face his aggressiveness through the defense mechanism of reaction formation, probably showing better adaptability. Mr. F appears more conscious of his “mediocrity,” but he doesn’t suffer too much for this, asserting that all the world is lousy and his family’s one is awful.

The therapist understands that to maintain a good climate during the therapeutic work it’s necessary to keep the family relationships, in particular the one with his mother, outside the setting. The patient feels too much anger toward the maternal figure, considering her unable to appreciate his qualities (and maybe also his pain) or, at most, an extremely anxious and oppressive person. He thinks he can relate to her only adopting a symmetrical attitude: he behaves with her in the same way she does with him, ignoring her. Only during some of Mr. F’s sessions does a more depressive (elaborative) organization appear, but it’s difficult for him to remain in this condition. The complex relationship with his mother develops as a sort of “refuge for the mind,” where Mr. F’s omnipotence triumphs and, in his fantasy, everything is allowed. Therefore, there’s a risk of isolation and consequent compromising of relationships with other people: it’s not by chance that Mr. F considers love relationships too demanding, if not actually useless, while he talks about presumed supposed contacts with who people who practice “partner swapping.”

In this year the work with Mr. F is sometimes characterized by the lack of a patient’s real motivation for change. His ambivalence in the relationships with main affective reference in the family (the mother above all) is reflected in the therapeutic work.

### Mr. F at the End of the Psychotherapy – Empirical Perspective

**FIGURE 3:**
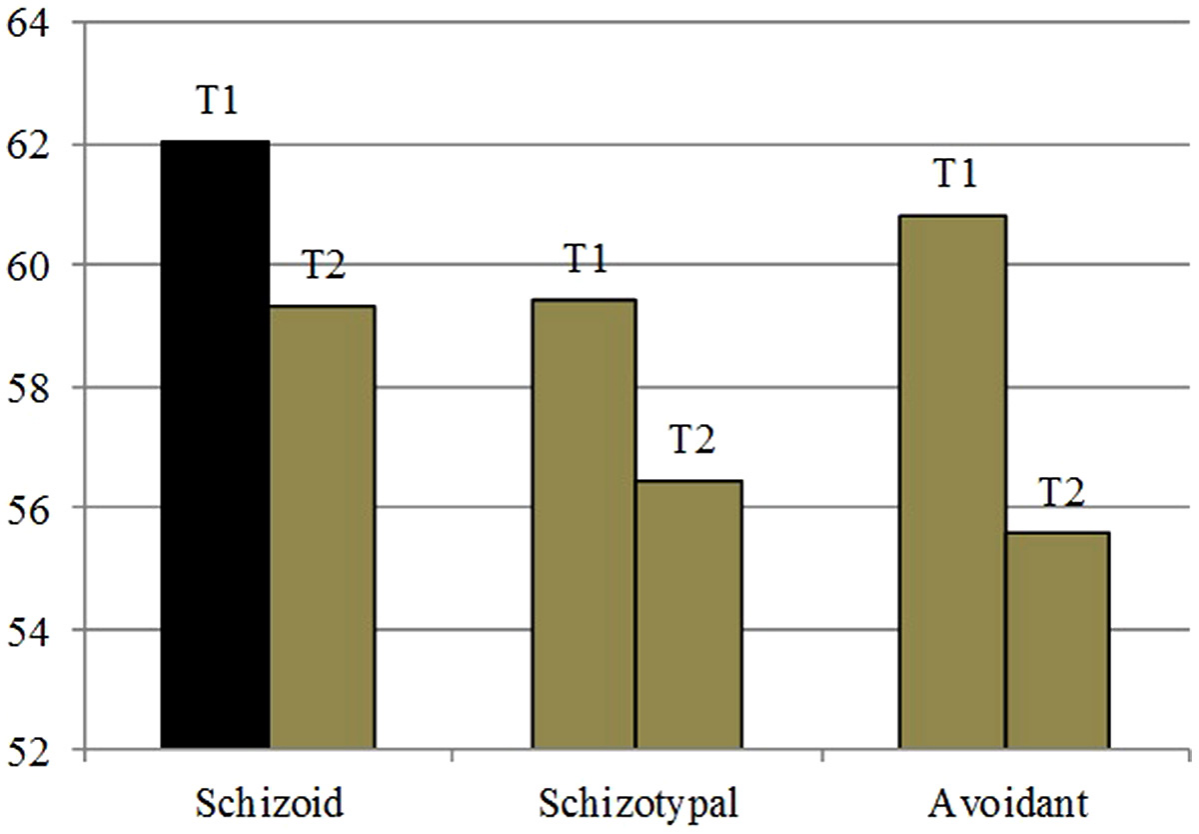
**SWAP-200 PD-T Scores at the beginning of the psychotherapy and after 1 year**.

After 1 year of treatment the HAM-D scored 4, indicating Mr. F totally remitted from the episode of MDD (F32.1). Also BDI II score decreases to 10, in a minimal range area. All PD scores were under 60, but remarkable was the tendency towards the Schizoid PD (*T* = 59,32), which was still quite firm at the end of the first year of the psychotherapy, the Schizotypal PD (*T* = 56,44), and the Avoidant PD (*T* = 55,57). However none of these values amounts to the level of Disorders (≥60), with a decrease in T2 of Schizoid trait and Avoidant Trait in particular. The High Functioning score is a bit higher, but remains under 50 (**Figure 3**).

**FIGURE 4:**
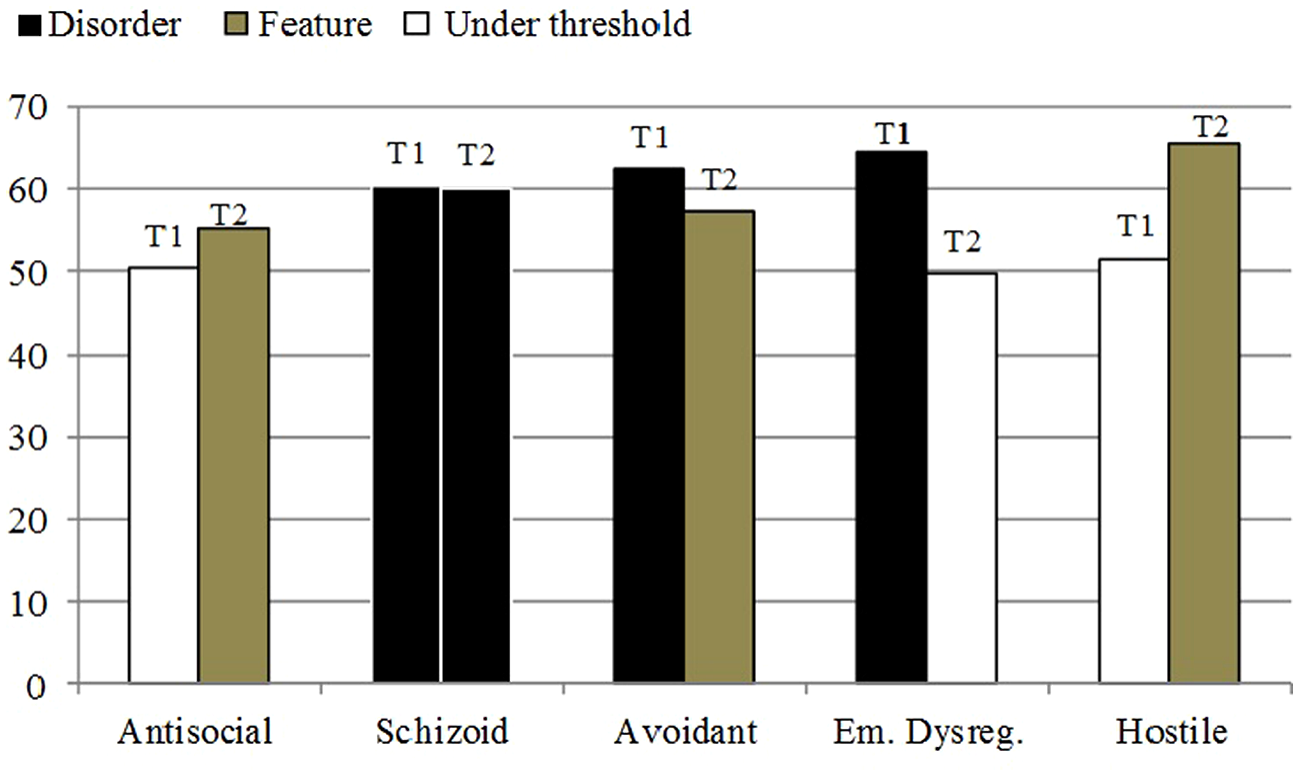
**SWAP-200 Q-Factor *T*-Scores at the beginning of the psychotherapy and after 1 year**.

Regarding Q scores profiles, the more important development from the beginning to the end of 1 year of treatment are related to the significant decrease of the Emotional Dysregulation PD (*T* = 49,85) and of the Avoidant PD (*T* = 57,25) and the significant increase of the Hostility PD (*T* = 65,48). At the end, the Schizoid PD remained stable (*T* = 60,22) (**Figure 4**).

**FIGURE 5:**
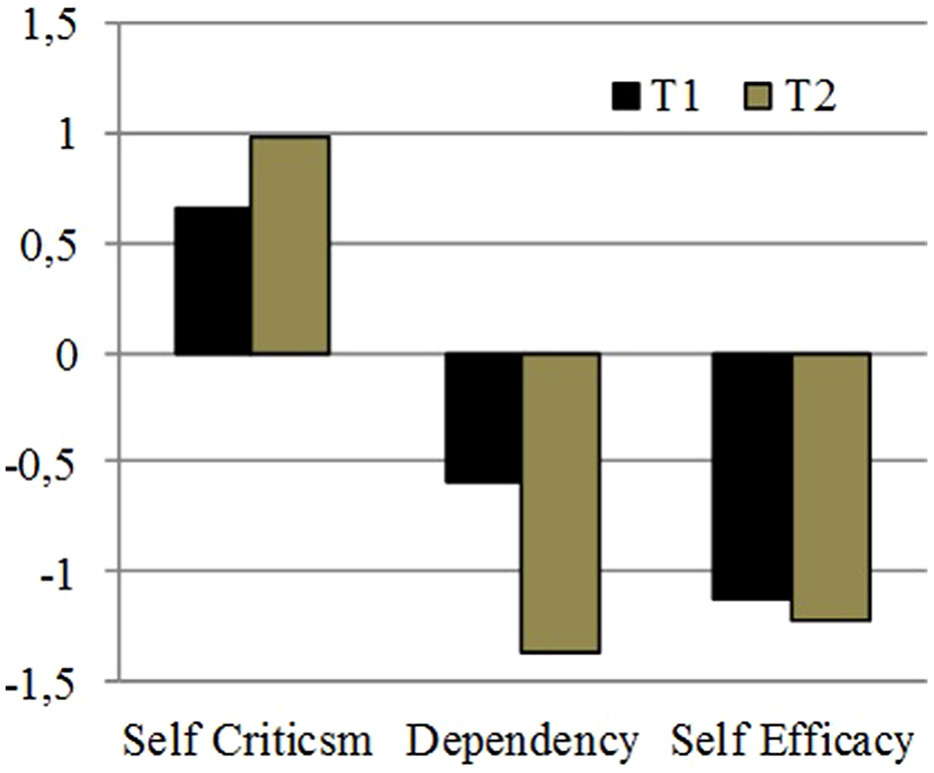
**The Depressive Experience Questionnaire (DEQ) dimensions at the beginning of the psychotherapy and after 1 year**.

The DEQ shows remarkable increase in the main dimensions following the tendence showed in T1. In particular, the depressive experience of Mr. F confirms an introjective profile (0,99), associated with the refusal of anaclitic relationships (–1,37) in a low self-efficacy perception (–1,23) (**Figure 5**).

## Discussion and Conclusion

Mr. F’s psychotherapy has shown good results concerning reduction of depressive symptomatology, according to important research examinated in scientific literature: in particular, the best outcome results are reported from the psychotherapy, also in psychodynamic framework, combinated with pharmacotherapy (Keller et al., 2000; Burnand et al., 2002; Cuijpers et al., 2014), as in clinical case study here presented. Even though symptomatic remission is considered a fundamental result for a successful therapy, the problem is to evaluate possible change in specific features of personality and mental functioning. Symptoms reduction itself does not say much about the nature of the therapeutic change (Horowitz, 1993; Blatt and Auerbach, 2003).

Starting from the clinical case study presented, it is possible to highlight some important change in personality configurations: in particular, a reduction of emotional dysregulation, dimensions which are more strongly linked to the depressive experience and to the emotional adaptation and regulation (Ehring et al., 2010; Carl et al., 2013), is notable (see SWAP-200 results). In a stable drug assumption during the year of the treatment (duloxetina, 60 mg die), this modification is very likely connected with psychotherapy work. The use of DEQ allows a deep analysis of depressive state: the patient appears to be characterized by a depression of introjective type, with predominant scores in autocritical aspects. Its prevalence is located in MMD (Blatt and Zuroff, 1992; Blatt and Levy, 1998; Blatt, 2004, 2008; Luyten et al., 2007). Mr. F presents some typical symptoms of MDD (American Psychiatric Association, 2000) at the beginning of the treatment: they decrease a lot after one year. On the other hand, in the PDM (PDM Task Force, 2006) attention is paid to the difference between patients with the MMD and patients with depressive personality. Some features of this kind of personality appear stable over the time in Mr. F: for example, important difficulties in relationships, with inclinations to perceive self as inadequate or refused by others, as well as typical defensive arrangements with turnover of the devaluation of the self or of the others associated with introjective or projective mechanism.

The verbal interactions with the psychotherapist analyzed by CCRT strengthened the hypothesis that the patient considers himself unable to receive the care of the others (Eckert et al., 1990). Anger is often directed towards people who live with the patient (the mother in particular) and, in this way, he avoids having strong feelings of inadequately and guilt. Since its outset – Abraham (1911) and Freud (1915) – psychoanalysis described this mental condition as typical of depression. In this perspective the increase in the hostile dimension (Q Factor/Style from 51.64 to 65.48) measured by SWAP-200 appears as a new strategy of the patient to externalize the anger preserving his inner balance. As underlined by Busch et al. (2004), management of anger and of narcissistic injury represents an important part within the psychodynamic psychotherapy of depression.

Also, the analysis of defenses leads supposing a change in the management of anger by Mr. F. The increase in the tendency to use defenses of the acting cluster between the second and the third phases of the treatment can be explained with his greater inclination to seek attention and help from others in order to demonstrate that nobody can be useful to him. In this way the hostility is addressed to the outside as the increase in the hostility dimension in SWAP-200 shows. On the other hand, the decrease in dysfunctional defenses matched with the statement of mature defenses, humor, and self-affirmation in particular, encourage the belief of a real different mental functioning of the patient after 1 year of treatment is present (Bond and Vaillant, 1986; Bond, 2004; Perry and Bond, 2012). The CCRT interactions in the third phase of the treatment lead to the same consideration: Mr. F seems more able to manage his anger and his emotions in general. In this perspective a remarkable decrease of emotional dysregulation dimension in SWAP-200 has to be borne in mind. Generally speaking, at the start of the psychotherapy the patient appeared to be antisocial and not so inclined to discuss his assumptions; the most frequently used defenses are those of denial which allow the patient to exclude from his awareness both the cognitive and the affective components of painful events and thoughts allowing in to get literally rid of them, and attributing to others or distorting their significance through ad hoc explanations and justifications. At the end of the therapy the patient seems more aware and less distressed to get rid of the painful aspects of his experience; as a matter of fact, we assist at a decline in the denial defenses and at an increase of mature defenses such as humor which allows Mr. F to ironize on the situation. Nevertheless, the ODF level suggests the patient endures in clinical ranges (Perry, 1991).

Another dimension emerging from SWAP-200 has to be considered with attention: the continuity of the prevalence of the Schizoid trait. In particular, while the PD scores go under threshold in T2, the *Q* scores preserve an elevation in T2Q Factors/Styles scores (60.44 in T1; 60.22 in T2). A part of literature confirms possible relationships between depressive disorders and some specific PDs – in particular Schizoid or Cluster A PDs and Avoidant PDs (Sato et al., 1994; Hirschfeld, 1999; Johnson et al., 2005), but also the description of the two different dimensions of depression by Blatt (Blatt, 1974; Cicchetti and Aber, 1986; Blatt and Homann, 1992; Blatt and Maroudas, 1992; Zuroff and Fitzpatrick, 1995) can be useful. In particular, it is possible to argue that two different basic personality configurations – anaclitic and introjective – are related to specific disordered behavior and the self-definitional (or introjective) developmental line is related to certain personality traits or disorders: among them Schizoid and Schizotypal Disorders (Blatt and Shichman, 1983; Ouimette and Klein, 1993; Ouimette et al., 1994). In the same way, it is possible to suppose that the avoidant trait (disorder) in Mr. F can be linked to fearful avoidant attachment (Levy et al., 1998; Meyer et al., 2001; Reis and Grenyer, 2002) as the relationship with the mother and the psychotherapist encourages to think.

The Mr. F case study describes how a good psychotherapeutic treatment in Depressive Disorder condition can develop. Indeed, the psychodynamic framework allows to show more clearly how the changes can happen. Even if the presence of the PDs might have a negative impact on the outcome of the treatment of depression (Newton-Howes et al., 2006, 2014), in the present case study the presence and variations of specific traits and features of personality were interpreted as a general adjustment of Mr. F concerning its “internal world” and its relations with others in an interpersonal perspective (Sullivan, 1953). As Blatt and Luyten (2009) and Luyten and Blatt (2013) suggested, self-definition and interpersonal relatedness in the developmental stages found two fundamental personality dimensions, useful for understanding pathological and normal condition and evaluating psychotherapy results outcomes (Blatt and Shahar, 2004; Blatt et al., 2007). In contrast with different profiles from SWAP-200, this consideration may explain the stability of introjective dimension in Mr. F over time and the greater difficulty in improving mature integration in his latent mental structures that presents an introjective profile (Werbart and Forsström, 2014). On the other hand, some modifications in the psychotherapeutic relationship, as the CCRT analysis shown, could be a good predictor for enhancing of the adaptive capacities (Blatt et al., 2010). In this perspective, this study highlights the usefulness of the psychodynamic treatment in a public setting. Even if there is an important limitation due to the duration of the treatment – only 1 year –, in Mr. F some change in terms of personality configuration and mental functioning are well recognizable. So, after this period of treatment, it is possible to affirm to have had a good results in term of *effectiveness* (Kendall et al., 2004; Lambert and Ogles, 2004): comparing his situation at the beginning of the psychotherapy, after one year the patient has shown a better awareness of his relationship style and of his inner conflicts, introducing new strategy of adaptation in his life. Obviously any generalizations should not be made and other clinical studies are welcome.

## Conflict of Interest Statement

The author declares that the research was conducted in the absence of any commercial or financial relationships that could be construed as a potential conflict of interest.

## References

[B1] Ablon J. S. Jones E. E. (1999). Psychotherapy process in the national institute of mental health treatment of depression collaborative research program. *J. Consult. Clin. Psychol.* 67 64–75 10.1037/0022-006X.67.1.64 10028210

[B2] Abraham K. (1911). “Notes on the psycho-analytical investigation and treatment of manic-depressive insanity and allied conditions,” in *Selected Papers of Karl Abraham* (1927) (London: Hogarth Press) 137–156.

[B3] Akkerman K. Lewin T. J. Carr V. J. (1999). Long-term changes in defense style among patients recovering from major depression. *J. Nerv. Ment. Dis.* 187 80–87 10.1097/00005053-199902000-00003 10067947

[B4] American Psychiatric Association. (2000). *Diagnostic and Statistical Manual of Mental Disorders DSM-IV-TR 4th Fourth Edition, Text Revision*. Washington, DC: American Psychiatric Association. 10.1176/appi.books.9780890423349

[B5] Arieti S. Bemporad J. (1978). *Severe and Mild Depression: The Psychotherapeutic Approach*. New York: Basic Books.

[B6] Asch S. S. (1966). Depression: three clinical variations. *Psychoanal. Study Child* 21 150–171. 5965392 10.1080/00797308.1966.11823256

[B7] Beck A. T. Steer R. A. Brown G. K. (1996). *Beck Depression Inventory,* 2nd Edn. San Antonio, TX: The Psychological Corporation.

[B8] Black D. Bell S. Hulbert J. Nasrallah A. (1988). The importance of axis II in patients with major depression: a controlled study. *J. Affect. Disord.* 14 115–122 10.1016/0165-0327(88)90053-5 2966824

[B9] Blatt S. J. (1974). Levels of object representation in anaclitic and introjective depression. *Psychoanal. Study Child* 29 107–157. 4445397

[B10] Blatt S. J. (2004). *Experiences of Depression. Theoretical, Clinical, and Research Perspectives.* Washington, DC: American Psychological Association. 10.1037/10749-000

[B11] Blatt S. J. (2008). *Polarities of Experiences. Relatedness and Self-definition in Personality Development, Psychopathology, and the Therapeutic Process*. Washington, DC: American Psychological Association. 10.1037/11749-000

[B12] Blatt S. J. Auerbach J. S. (2003). Psychodynamic measures of therapeutic change. *Psychoanal. Inquiry* 23 268–307 10.1080/07351692309349034

[B13] Blatt S. J. Besser A. Ford R. Q. (2007). Two primary configurations of psychopathology and change in thought disorder in long-term intensive inpatient treatment of seriously disturbed young adult. *Am. J. Psychiatry* 164 1561–1567 10.1176/appi.ajp.2007.05111853 17898348

[B14] Blatt S. J. D’Aﬄitti J. P. Quinlan D. M. (1976). Experiences of depression in normal young adults. *J. Abnorm. Psychol.* 85 383–389 10.1037/0021-843X.85.4.383 956505

[B15] Blatt S. J. Homann E. (1992). Parent–child interaction in the etiology of dependent and self-critical depression. *Clin. Psychol. Rev.* 12 47–91 10.1016/0272-7358(92)90091-L

[B16] Blatt S. J. Levy K. N. (1998). “A psychodynamic approach to the diagnosis of psychopathology,” in *Making Diagnosis Meaningful: Enhancing Evaluation and Treatment of Psychological Disorders* ed. Barron J. W. (Washington, DC: American Psychological Association) 73–109.

[B17] Blatt S. J. Luyten P. (2009). A structural-developmental psychodynamic approach to psychopathology: two polarities of experience across the life span. *Dev. Psychopathol.* 21 793–814 10.1017/S0954579409000431 19583884

[B18] Blatt S. J. Maroudas C. (1992). Convergence of psychoanalytic and cognitive behavioral theories of depression. *Psychoanal. Psychol.* 9 157–190 10.1037/h0079351

[B19] Blatt S. J. Quinlan D. M. Pilkonis P. A. Shea M. T. (1995). Impact of perfectionism and need for approval on the brief treatment of depression: the national institute of mental health treatment of depression collaborative research program revisited. *J. Consult. Clin. Psychol*. 63 125–132 10.1037/0022-006X.63.1.125 7896977

[B20] Blatt S. J. Sanislow C. A. III Zuroff D. C. Pilkonis P. A. (1996). Characteristics of effective therapists: further analyses of data from the National Institute of Mental Health Treatment of Depression Collaborative Research Program. *J. Consult. Clin. Psychol.* 64 1276–1284 10.1037//0022-006x.64.6.1276 8991314

[B21] Blatt S. J. Shahar G. (2004). Stability of the patient-by-treatment interaction in the Menninger Psychotherapy Research Project. *Bull. Menninger. Clin.* 68 23–38 10.1521/bumc.68.1.23.27733 15113032

[B22] Blatt S. J. Shichman S. (1983). Two primary configurations of psychopathology. *Psychoanal. Contemp. Thought* 6 187–254 10.1037/11749-006

[B23] Blatt S. J. Zuroff D. C. (1992). Interpersonal relatedness and self-definition: two prototypes for depression. *Clin. Psychol. Rev.* 12 527–562 10.1016/0272-7358(92)90070-O

[B24] Blatt S. J. Zuroff D. C. (2005). Empirical evaluation of the assumptions in identifying evidence based treatments in mental health. *Clin. Psychol. Rev.* 25 459–486 10.1016/j.cpr.2005.03.001 15893862

[B25] Blatt S. J. Zuroff D. C. Bondi C. M. Sainslow C. A. (2000). Short and long-term effects of medication and psychotherapy in the brief treatment of depression: further analyses of data from the NIMH TDCRP. *Psychother. Res.* 10 215–234 10.1080/713663676 22239698

[B26] Blatt S. J. Zuroff D. C. Hawley L. L. Auerbach J. S. (2010). Predictors of sustained therapeutic change. *Psychother. Res.* 20 37–54 10.1080/10503300903121080 19757328

[B27] Blom M. B. J. Spinhoven P. Hoffman T. Jonker K. Hoencamp E. Haffmans P. M. J. (2007). Severity and duration of depression, not personality factors, predict short term out come in the treatment of major depression. *J. Affect. Disord.* 104 119–126 10.1016/j.jad.2007.03.010 17467059

[B28] Bond M. (2004). Empirical studies of defense style: relationships with psychopathology and change. *Harv. Rev. Psychiatry* 12 263–278 10.1080/10673220490886167 15590575

[B29] Bond M. Perry J. C. (2004). Long-term changes in defense styles with psychodynamic psychotherapy for depressive, anxiety, and personality disorders. *Am. J. Psychiatry* 161 1665–1671 10.1176/appi.ajp.161.9.1665 15337658

[B30] Bond M. Vaillant J. S. (1986). An empirical study of the relationship between diagnosis and defense style. *Arch. Gen. Psychiatry* 43 285–288 10.1001/archpsyc.1986.01800030103012 3954550

[B31] Burnand Y. Andreoli A. Kolatte E. Venturini A. Rosset N. (2002). Psychodynamic psychotherapy and clomipramine in the treatment of major depression. *Psychiatr Serv.* 53 585–590 10.1176/appi.ps.53.5.585 11986508

[B32] Busch F. N. Rudden M. Shapiro T. (2004). *Psychodynamic Treatment of Depression.* Washington, DC: American Psychiatric Publishing.

[B33] Calati R. Oasi O. De Ronchi D. Serretti A. (2010), The use of the defence style questionnaire in major depressive and panic disorders: a comprehensive meta-analysis. *Psychol. Psychother*. 83 1–13 10.1348/147608309X464206 19671241

[B34] Carl J. R. Soskin D. P. Kerns C. Barlow D. H. (2013). Positive emotion regulation in emotional disorders: a theoretical review. *Clin. Psychol. Rev.* 33 343–360 10.1016/j.cpr.2013.01.003 23399829

[B35] Carter J. D. Luty S. E. McKenzie J. M. Mulder R. T. Frampton C. M. Joyce P. R. (2011). Patient predictors of response to cognitive behaviour therapy and interpersonal psychotherapy in a randomised clinical trial for depression. *J. Affect. Disord.* 128 252–261 10.1016/j.jad.2010.07.002 20674982

[B36] Casey P. Meagher D. Butler E. (1996). Personality, functioning, and recovery from major depression. *J. Nerv. Ment. Dis.* 184 240–245 10.1097/00005053-199604000-00007 8604034

[B37] Castelnuovo G. (2010). Empirically supported treatments in psychotherapy: towards an evidence-based or evidence-biased psychology in clinical settings? *Front.* *Psychol.* 1:27. 10.3389/fpsyg.2010.00027 PMC3153746 21833197

[B38] Charney D. S. Nelson J. C. Mac Quinlan D. M. (1981). Personality traits and disorder in depression. *Am. J. Psychiatry* 138 1601–1604 10.1176/ajp.138.12.1601 7304794

[B39] Cicchetti D. Aber L. J. (1986). “Early precursors to later depression: an organizational perspective,” in *Advances in Infancy* Vol. 4 eds Lipsitt L. Rovee-Collier C. (Norwood, NJ: Ablex) 87–137.

[B40] Cuijpers P. Sijbrandij M. Koole S. L. Andersson G. Beekman A. T. Reynolds C. F. (2014). Adding psychotherapy to antidepressant medication in depression and anxiety disorders: a meta-analysis. *World Psychiatry* 13 56–67 10.1002/wps.20089 24497254 PMC3918025

[B41] Diguer L. Barber J. P. Luborsky L. (1993). Three concomitants: personality disorders, psychiatric severity and outcome of dynamic psychotherapy of major depression. *Am. J. Psychiatry* 150 1246–1248 10.1176/ajp.150.8.1246 8328572

[B42] Eckert R. Luborsky L. Barber J. Crits-Christoph P. (1990). “The narratives and CCRT’s of clients with major depression,” in *Understanding Transference: The CCRT Method* eds Luborsky L. Crits-Christoph P. (New York: Basic Book) 222–234.

[B43] Ehring T. Tuschen-Caffier B. Schnülle J. Fischer S. Gross J. J. (2010). Emotion regulation and vulnerability to depression: spontaneous versus instructed use of emotion suppression and reappraisal. *Emotion* 10 563–572 10.1037/a0019010 20677873

[B44] Elkin I. Gibbons R. D. Shea M. T. Shaw B. F. (1996). Science is not a trial (but it can sometimes be a tribulation). *J. Consult. Clin. Psychol.* 64 92–103 10.1037/0022-006X.64.1.92

[B45] Elkin I. Parloff M. B. Hadley S. W. Autry J. H. (1985). NIMH treatment of depression collaborative research program. Background and research plan. *Arch. Gen. Psychiatry* 42 305–316 10.1001/archpsyc.1985.01790260103013 2983631

[B46] Elkin I. Shea M. T. Watkins J. T. Imber S. D. Sotsky S. M. Collins J. F. (1989). National institute of mental health treatment of depression collaborative research program: general effectiveness of treatments. *Arch. Gen. Psychiatry* 46 971–982 10.1001/archpsyc.1989.01810110013002 2684085

[B47] First M. B. Spitzer R. L. Gibbon M. Williams J. B. W. (1997a). *SCID-I Structured Clinical Interview for DSM-IV Axis I Disorders*. Washington, DC: American Psychiatric Press.

[B48] First M. B. Gibbon M. Spitzer R. L. Williams J. B. W. Benjamin L. S. (1997b). *SCID-II Structured Clinical Interview for DSM-IV Axis II Disorders*. Washington, DC: American Psychiatric Press.

[B49] Fonagy P. (1993). Psychoanalytic and empirical approaches to developmental psychopathology: can they be usefully integrated? *J. R. Soc. Med.* 86 577–581. 7693943 PMC1294137

[B50] Fonagy P. Moran G. (1993). “Selecting single case research designs for clinicians,” in *Handbook of Psychodynamic Treatment Research* eds Miller N. Luborsky L. Barber J. P. Docherty J. P. (New York: Basic Book) 62–95.

[B51] Freud S. (1915). “Mourning and Melancholia,” in *The Standard Edition of the Complete Psychological Works of Sigmund Freud* Vol. XIV ed. Strachey J. (London: The Hogarth Press and the Institute of Psycho-analysis) 243–257.

[B52] Gabbard G. O. (2000). *Psychodynamic Psychiatry in Clinical Practice*. Washington, DC: American Psychiatric Press.

[B53] Gallagher-Thompson D. Steffen A. M. (1994). Comparative effects of cognitive-behavioral and brief psychodynamic psychotherapies for depressed family caregivers. *J. Consult. Clin. Psychol.* 62 543–549 10.1037/0022-006X.62.3.543 8063980

[B54] Hamilton M. (1960). A rating scale for depression. *J. Neurol. Neurosurg. Psychiatry* 23 56–62 10.1136/jnnp.23.1.56 14399272 PMC495331

[B55] Hilsenroth M. J. Ackerman S. J. Blagys M. D. Baity M. R. Mooney M. A. (2003). Short-term psychodynamic psychotherapy for depression: an examination of statistical, clinically significant, and technique-specific change. *J. Nerv. Ment. Dis.* 191 349–357 10.1097/01.NMD.0000071582.11781.67 12826915

[B56] Hirschfeld R. M. A. (1999). Personality disorders and depression: comorbidity. *Depress. Anxiety* 10 142–146 10.1002/(SICI)1520-6394(1999)10:4<142::AID-DA2>3.0.CO;2-Q 10690575

[B57] Hirschfeld R. M. A. Klerman G. L. Clayton P. J. Keller M. B. (1983). Personality and depression. *Empirical. Findings. Arch. Gen. Psychiatry* 40 993–998 10.1001/archpsyc.1983.01790080075010 6615162

[B58] Hollon S. D. Thase M. E. Markowitz J. C. (2002). Treatment and prevention of depression. *Psychol. Sci. Public Interest* 3 39–77 10.1111/1529-1006.00008 26151569

[B59] Horowitz M. (1993). “Personality structure and the process of change during psychoanalysis,” in *Psychic Structure and Psychic Change: Essays in Honor of Robert S. Wallerstein, M.D.* eds Horowitz M. Kernberg O. F. Weinshel E. M. (Madison, CT: International University Press) 1–28.

[B60] Jacobson E. (1971). *Depression: Comparative Studies of Normal, Neurotic, and Psychotic Conditions*. New York: International Universities Press.

[B61] Jacobson N. S. Hollon S. D. (1996). Cognitive-behavior therapy versus pharmacotherapy: now that the juri’s returned its verdict, it’s time to present the rest of the evidence. *J. Consult. Clin. Psychol.* 64 74–80 10.1037/0022-006X.64.1.74 8907086

[B62] Jakobsen J. C. Hansen J. L. Storeb ø O. J. Simonsen E. Gluud C. (2011a). The effects of cognitive therapy versus ‘treatment as usual’ in patients with major depressive disorder. *PLoS ONE* 6:e22890 10.1371/journal.pone.0022890 PMC3150380 21829664

[B63] Jakobsen J. C. Hansen J. L. Simonsen E. Gluud C. (2011b). The effect of interpersonal psychotherapy and other psychodynamic therapies versus ‘treatment as usual’ in patients with major depressive disorder. *PLoS ONE* 6:e19044 10.1371/annotation/d74286e2-e872-4a7b-87d1-dfa230ff612d PMC3083428 21556370

[B64] Johnson J. G. Cohen P. Kasen S. Brook J. S. (2005). Personality disorder traits associated with risk for unipolar depression during middle adulthood. *Psychiatry Res.* 136 113–121 10.1016/j.psychres.2005.02.007 16091291

[B65] Kächele H. Schachter J. Thomä H. (eds) (2009). *From Psychoanalytic Narrative to Empirical Single Case Research.* New York: Routledge.

[B66] Kazdin A. E. (1982). *Single Case Research Designs*. New York: Oxford University Press.

[B67] Keller M. B. McCullough J. P. Klein D. N. Arnow B. Dunner D. L. Gelenberg A. J. (2000). A comparison of nefazodone, the cognitive behavioral-analysis system of psychotherapy, and their combination for the treatment of chronic depression. *N. Engl. J. Med.* 342 1462–1470 10.1056/NEJM200005183422001 10816183

[B68] Kendall P. C. Holmbeck G. Verduin T. (2004). “Methodology, design and evaluation in psychotherapy research,” in *Bergin and Garfield’s Handbook of Psychotherapy and Behavior Change* ed. Lambert M. J. (New York: John Wiley & Sons) 16–43.

[B69] Klein M. H. Wonderlich S. Shea M. T. (1993). “Model of relationships between personality and depression: toward a framework for theory and research,” in *Personality and Depression: A Current View* eds Klein M. H. Kupfer D. J. Shea M. T. (New York: Guilford) 1–54.

[B70] Koenigsberg H. W. Kaplan R. D. Gilmore M. M. Cooper A. M. (1985). The relationship between syndrome and personality disorder in DSM-II: experience with 2.462 patients. *Am. J. Psychiatry* 142 207–212 10.1176/ajp.142.2.207 3970245

[B71] Lambert M. J. Ogles B. M. (2004). “The efficacy and effectiveness of psychotherapy,” in *Bergin and Garfield’s Handbook of Psychotherapy and Behavior Change* ed Lambert M. J. (New York: John Wiley & Sons) 139–193.

[B72] Levy K. N. Blatt S. J. Shaver P. R. (1998). Attachment styles and parental representations. *J. Pers. Soc. Psychol.* 74 407–419 10.1037/0022-3514.74.2.407

[B73] Levy R. A. Ablon J. S. Kächele H.(eds) (2012). *Psychodynamic Psychotherapy Research. Evidence-Based Practice and Practice-Based Evidence.* New York: Springer 10.1007/978-1-60761-792-1

[B74] Luborsky L. (1984). *Principles of Psychoanalytic Psychotherapy: A Manual for Supportive-Expressive Treatment.* New York: Basic Books.

[B75] Luyten P. (2014). Finally moving beyond the horse race: CBT and psychodynamic therapy equally effective for depression. *Evid. Based Mental. Health* 17 118 10.1136/eb-2014-101770 PMC13404033 25326494

[B76] Luyten P. Blatt S. J. (2012). Psychodynamic treatment of depression. *Psychiatr. Clin. N. Am.* 35 111–129 10.1016/j.psc.2012.01.001 22370494

[B77] Luyten P. Blatt S. J. (2013). Interpersonal relatedness and self-definition in normal and disrupted personality development: retrospect and prospect. *Am. Psychol.* 68 172–183 10.1037/a0032243 23586492

[B78] Luyten P. Sabbe B. Blatt S. J. Meganck S. Jansen B. De Grave C. (2007). Dependency and Self-criticism: relationship with major depressive disorder, severity of depression, and clinical presentation. *Depress. Anxiety* 24 586–596 10.1002/da.20272 17143851

[B79] Margison F. R. Barkham M. Evans C. McGrath G. Clark J. M. Audin K. (2000). Measurement and psychotherapy: evidence-based practice and practice-based evidence. *Br. J. Psychiatry* 177 123–130 10.1192/bjp.177.2.123 11026951

[B80] Meyer B. Pilkonis P. A. Proietti J. M. Heape C. L. Egan M. (2001). Attachment styles and personality disorders as predictors of symptom course. *J. Person. Disord.* 15 371–389 10.1521/pedi.15.5.371.19200 11723873

[B81] Moran G. S. Fonagy P. (1987). Psychoanalysis and diabetic control: a single-case study. Br*. J. Med. Psychol.* 60 357–372 10.1111/j.2044-8341.1987.tb02755.x 3426974

[B82] Mulder R. T. (2002). Personality pathology and treatment outcome in major depression: a review. *Am. J. Psychiatry* 159 359–371 10.1192/bjp.189.2.186b 11869996

[B83] Mullen L. S. Blanco C. Vaughan S. C. Vaughan R. Roose S. P. (1999). Defense mechanisms and personality in depression. *Depress. Anxiety* 10 168–174 10.1002/SICI1520-6394 10690578

[B84] Newton-Howes G. Tyrer P. Johnson T. (2006). Personality disorder and the outcome of depression: meta-analysis of published studies. *Br. J. Psych.* 188 13–20 10.1192/bjp.188.1.13 16388064

[B85] Newton-Howes G. Tyrer P. Johnson T. Mulder R. Kool S. Dekker J. (2014). Influence of personality on the outcome of treatment in depression: systematic review and meta-analysis. *J. Pers. Disord.* 28 577–593 10.1521/pedi_2013_27_070 24256103

[B86] O’Leary D. Costello F. (2001). Personality and outcome in depression: an 18-month prospective follow-up study. *J. Affect. Disord.* 63 67–78 10.1016/S0165-0327(00)00159-2 11246082

[B87] Ouimette P. C. Klein D. N. (1993). “Convergence of psychoanalytic and cognitive-behavioral theories of depression: a review of the empirical literature and new data on Blatt’s and Beck’s models,” in *Psychoanalytic Perspectives on Psychopathology* eds Masling J. Bornstein R. (Washington, DC: American Psychological Association) 191–223.

[B88] Ouimette P. C. Klein D. N. Anderson R. Riso L. P. Lizardi H. (1994). Relationship of sociotropy/autonomy and dependency/self-criticism to DSM-III-R personality disorders. *J. Abnorm. Psychol.* 103 743–749 10.1037/0021-843X.103.4.743 7822576

[B89] Patience D. A. McGuire R. J. Scott A. I. Freeman C. P. (1995). The edinburgh primary care depression study: personality disorder and outcome. *Br. J. Psychiatry* 167 324–330 10.1192/bjp.167.3.324 7496640

[B90] PDM Task Force. (2006). *Psychodynamic Diagnostic Manual (PDM).* Silver Spring, MD: Alliance of Psychoanalytic Organizations.

[B91] Peeters F. Huibers M. Roelofs J. van Breukelen G. Hollon S. D. Markowitz J. C. (2013). The clinical effectiveness of evidence-based interventions for depression: a pragmatic trial in routine practice. *J. Affect. Disord.* 145 349–355 10.1016/j.jad.2012.08.022 22985486

[B92] Perry J. C. (1991). *Defense Mechanisms Rating Scale*. Boston: Cambridge Hospital – Harvard Medical School.

[B93] Perry J. C. Bond M. (2012). Change in defense mechanisms during long-term dynamic psychotherapy and five-year outcome. *Am. J. Psychiatry* 169 916–925 10.1176/appi.ajp.2012.11091403 22885667

[B94] Perry J. C. Hoglend P. Shear K. Vaillant G. E. Horowitz M. Kardos M. E. (1998). Field trial of a diagnostic axis for defense mechanisms for DSM-IV. *J. Pers. Disord.* 12 56–68 10.1521/pedi.1998.12.1.56 9573520

[B95] Perry J. C. Kardos M. E. (1995). “A review of the Defense Mechanism Rating Scale,” in *Ego defenses: Theory and measurement* eds Conte H. R. Plutchick R. (New York: John Wiley & Sons) 283–299.

[B96] Perry J. C. Kardos M. E. Pagano C. J. (1993). “The study of defenses in psychotherapy using the Defense Mechanism Rating Scale DMRS,” in *The Concept of Defense Mechanisms in Contemporary Psychology: Theoretical, Research and Clinical Perspectives* eds Hentschel U. Ehlers W. (New York: Springer) 122–132.

[B97] Pfohl B. Black D. W. Noyes R. Coryell W. H. Barrash J. (1990). “Axis I / Axis II comorbidity findings: implications for validity,” in *Personality Disorders: New Perspectives on Diagnostic Validity* ed. Oldham J. M. (Washington, DC: American Psychiatric Association Press) 147–161.

[B98] Pfohl B. Stangl D. Zimmermann M. (1984). The implications of DSM-III personality disorders for patients with major depression. *J. Affect. Disord.* 7 309–318 10.1016/0165-0327(84)90052-1 6241212

[B99] Pilkonis P. A. Frank E. (1988). Personality pathology in recurrent depression: nature, prevalence, and relationship to treatment response. *Am. J. Psychiatry* 145 435–441 10.1176/ajp.145.4.435 3348446

[B100] Porcerelli J. H. Dauphin V. B. Ablon J. S. Leitman S. Bambery M. (2007). Psychoanalysis with avoidant personality disorder: a systematic case study. *Psychotherapy (Chic)* 44 1–13 10.1037/0033-3204.44.1.1 22122163

[B101] Power M. (ed.) (2013). *The Wiley Blackwell Handbook of Mood Disorders,* 2nd Edn. Chichester: Wiley-Blackwell. 10.1002/9781118316153

[B102] Quilty L. C. McBride C. Bagby R. M. (2008). Evidence for the cognitive mediational model of cognitive behavioral therapy for depression. *Psychol. Med.* 38 1531–1541 10.1017/S0033291708003772 18578895

[B103] Ramana R. Paykel E. S. Cooper Z. Hayhurst H. Saxty M. Surtees P. G. (1995). Remission and relapse in major depression: a two year prospective follow-up study. *Psychol. Med.* 25 1161–1170 10.1017/S0033291700033134 8637946

[B104] Reis S. Grenyer B. F. S. (2002). Pathways to anaclitic and introjective depression. *Psychol. Psychother.* 75 445–459 10.1348/147608302321151934 12626134

[B105] Roth A. Fonagy P. (1996). *What Works for Whom? A Review of Psychotherapy Research*. New York: Guilford Press.

[B106] Rush A. J. Thase M. E. (1999). “Psychotherapies for depressive disorders: a review,” in *Depressive Disroders* eds May M. Sartorius N. (New York: John Wiley & Sons) 161–206 10.1002/0470842342.ch3

[B107] Sato T. Sakado K. Sato S. Morikawa T. (1994). Cluster a personality disorder: a marker of worse treatment outcome of major depression? *Psychiatry Res*. 53 153–159 10.1016/0165-1781(94)90106-6 7824675

[B108] Serretti A. Calati R. Oasi O. De Ronchi D. Colombo C. (2007). Dissecting the determinants of depressive disorders outcome: an in depth analysis of two clinical cases. *Ann. Gen. Psychiatry* 7 6–5 10.1186/1744-859X-6-5 PMC1797808 17286859

[B109] Shapiro D. A. Barkham M. Rees A. Hardy G. E. Reynolds S. Startup M. (1994). Effects of treatment duration and severity of depression on the effectiveness of cognitive-behavioral and psychodynamic-interpersonal psychotherapy. *J. Consult. Clin. Psychol.* 62 522–534 10.1037/0022-006X.62.3.522 8063978

[B110] Shapiro D. A. Rees A. Barkham M. Hardy G. Reynolds S. Startup M. (1995). Effects of treatment duration and severity of depression on the maintenance of gains after cognitive-behavioral and psychodynamic-interpersonal psychotherapy. *J. Consult. Clin. Psychol.* 63 378–387 10.1037/0022-006X.63.3.378 7608350

[B111] Shea M. T. Glass D. Pilkonis P. A. Watkins J. Docherty J. P. (1987). Frequency and implications of personality disorders in a sample of depressed outpatients. *J. Pers. Disord.* 1 27–42 10.1521/pedi.1987.1.1.27

[B112] Shedler J. (2002). A new language for psychoanalytic diagnosis. *J. Am. Psychoanal Assoc.* 50 429–456 10.1177/00030651020500022201 12206539

[B113] Shedler J. Westen D. Lingiardi V. (2014). *La Valutazione Della Personalità Con la SWAP-200*. Milano: Raffaello Cortina Editore.

[B114] Stone L. (1986). Psychoanalytic observations on the pathology of depressive illness: selected spheres of ambiguity or disagreement. *J. Am. Psychoanal. Assoc.* 34 329–362 10.1177/000306518603400205 3722700

[B115] Sullivan H. S. (1953). *The Interpersonal Theory of Psychiatry*. New York: Norton.

[B116] Wallerstein R. S. (1995). *The Talking Cures. The Psychoanalyses and the Psychotherapies*. New Haven: Yale University Press.

[B117] Werbart A. Forsström D. (2014). Changes in anaclitic-introjective personality dimensions, outcomes and psychoanalytic technique: a multi-case study. *Psychoanal. Psychother.* 28 397–410 10.1080/02668734.2014.964295

[B118] Westen D. Shedler J. (1999a). Revising and assessing axis II, part 1: developing a clinically and empirically valid assessment method. *Am. J. Psychiatry* 156 258–272. 9989563 10.1176/ajp.156.2.258

[B119] Westen D. Shedler J. (1999b). Revising and assessing axis II, part 2: toward an empirically based and clinically useful classification of personality disorders. *Am. J. Psychiatry* 156 273–285. 9989564 10.1176/ajp.156.2.273

[B120] Widiger T. A. (1993). “Personality and depression: assessment issues,” in *Personality and Depression: A Current View* eds Klein M. H. Kupfer D. J. Shea M. T. (New York: Guilford) 77–118.

[B121] Zuroff D. C. Fitzpatrick D. (1995). Depressive personality styles: implications for adult attachment. *Person. Individ. Diff*. 18 253–265 10.1016/0191-8869(94)00136-G

